# Epigenetic responses to abiotic stresses during reproductive development in cereals

**DOI:** 10.1007/s00497-018-0343-4

**Published:** 2018-06-26

**Authors:** Kevin Begcy, Thomas Dresselhaus

**Affiliations:** Cell Biology and Plant Biochemistry, Biochemie-Zentrum Regensburg, University of Regensburg, 93053 Regensburg, Germany

**Keywords:** Abiotic stress, Germline, Fertilization, Seed development, DNA methylation, Histone modification, Small RNAs, Maize, Rice, Wheat

## Abstract

**Key message:**

**Overview of current understanding of epigenetic alterations after abiotic stresses during reproductive development in cereals.**

**Abstract:**

Abiotic stresses, including heat, drought, cold, flooding, and salinity, negatively impact crop productivity. Various stages during reproductive development are especially sensitive to environmental stresses, which may lead to complete sterility and severe yield losses. Plants exhibit diverse responses to ameliorate stress damage. Changes in DNA methylation, histone modification as well as regulation of small RNA and long noncoding RNA pathways have been shown to represent key modulators in plant stress responses. During reproductive development in cereals, various protein complexes controlling histone and DNA methylation have been identified, revealing conserved and novel mechanisms regulating abiotic stress responses in cereals and other plant species. New findings highlight the role of transposable elements during stress periods. Here, we review our current understanding of epigenetic stress responses during male and female gametophyte formation (germline development), fertilization, early seed devolvement, and seed maturation in cereals. An integrative model of epigenetic responses during reproductive development in cereals is proposed, emphasizing the role of DNA methylation and histone modifications during abiotic stresses.

## Introduction

With the human population continuously growing, the demand for food supply is increasing exponentially. Unfortunately, production of cereal grains, including some of the most important crops such as maize, wheat, rice, barley, rye, and oats, is highly vulnerable to abiotic stresses (Barnabas et al. 2008; Begcy and Walia 2015a, b; Chen et al. 2016; Ejaz and von Korff 2017; Folsom et al. 2014) and thus severely threatens the production of sufficient food for human and animal consumption. Abiotic stresses, such as cold, heat, salinity, drought, and flooding, which are associated with global climate change, negatively affect plant growth and development and thus represent a major obstacle for sufficient crop production. Reproductive development is especially sensitive to certain stresses, which may cause sterility and losses in grain yield, directly impacting productivity.

Cereals have developed different mechanisms to survive to both constantly changing and extreme environmental conditions (Barnabas et al. 2008; Bartels and Sunkar 2005; Basu et al. 2016; Begcy et al. 2011; Fahad et al. 2017; Mattiello et al. 2014). However, the responses of cereals to environmental stresses largely depend on their specific developmental stage. For instance, common responses during vegetative development include reduced light perception and perturbations in processes associated with carbon assimilation and transpiration (Barnabas et al. 2008). On the other hand, during reproductive development more detrimental responses are observed including male and female gametophyte malformation, sterility, seed abortion, as well as shortening of developmental phases and transitions. In contrast to vegetative development, in which stress periods can be tolerated to a certain extent, if stresses occur at critical stages during reproductive development such as male and female gametophyte formation (germline development), fertilization, early seed devolvement, and seed maturation, negative effects in grain yield are commonly observed.

Reproductive development begins with the formation of flowers. This process, which includes the transition of vegetative to flower meristems, and which is strongly influenced by environmental factors, has been nicely reviewed elsewhere (e.g., Hyun et al. 2017) and, therefore, is not the focus of this review. Therefore, here we are discussing the successive reproductive stages in cereals. Inside flower organs, male gametophytes (pollen) and female gametophytes (embryo sacs) are produced in anthers and ovules, respectively. While in most cereals (i.e., in rice, wheat, and barley) ovary and anthers are located in the same flower, species such as maize generate male flowers containing anthers as an apical tassel inflorescence and female flowers containing ovaries from axillary meristems forming cobs. During the processes of micro- and megasporogenesis including meiosis as well as micro- and megagametogenesis, both male and female gametophytes form gametes. In cereals, like in many other flowering plant species, pollen at the mature tri-cellular stage contains two sperm cells, while the embryo sac harbors a haploid egg and a di-haploid central cell (Zhou et al. 2017). After maturity, pollen grains are released from anthers and transferred to the stigma consisting of multicellular papilla hairs. Pollen grains adhere, hydrate, germinate, and penetrate into papilla hairs to grow toward one of the two transmitting tracts. Transmitting tracts serve as highways toward the ovule and ultimately guide pollen tubes toward the micropylar region of the female gametophyte (Lausser et al. 2010). Short-range guidance is necessary to lure the pollen tube toward the egg apparatus (egg cell and two synergid cells). After reaching its final destination, two sperm cells are released. One of the sperm cells fuses with the egg cell and the other one fuses with the central cell to produce the embryo and the endosperm, respectively (Bleckmann et al. 2014). These coordinated and well-established processes are altered or disrupted when plants are submitted to abiotic stresses.

Recent evidences propose that DNA methylation-, histone modification-, small regulatory RNA (sRNA)- and long noncoding RNA (lncRNA)-associated regulatory pathways are affected during plant development under stress conditions. These include stages of reproductive development such as gametogenesis and seed development (Begcy and Walia 2015a, b; Chen et al. 2016; Folsom et al. 2014; Zemach et al. 2010). In cereal reproductive development, it was shown that DNA methylation is a fundamental step for transcriptional repression of transposable elements (TEs) and regulation of gene expression (Huh et al. 2007; Zemach et al. 2010), a mechanism well described during vegetative development in other plant species. DNA methylation occurs at cytosine residues within three different sequence contexts (CG, CHG, and CHH). While methylation at CHG and CHH sites is predominantly found in TEs, CG methylation occurs in TEs and protein-encoding genes (Cui and Cao 2014; He et al. 2010; Lanciano and Mirouze 2017; Pikaard and Mittelsten Scheid 2014; Xia et al. 2017; Zemach et al. 2010). Chromatin marks and chromatin modifying enzymes also play a critical role by regulating gene expression under both optimal and sub-optimal conditions. The central unit of chromatin is the nucleosome, which is formed by an octamer of four core histones (H2A, H2B, H3, and H4,) wrapped by 147 base pairs of DNA. Histone N-terminal tails are essential for their regulation, since a large number and type of modified residues are located in this region (Asensi-Fabado et al. 2017; Kouzarides 2007). In cereals as well as in other plant species, histone modifications at these sites including methylation, acetylation, phosphorylation, ubiquitination, and SUMOylation are the most studied. Different residues, typically mono-, di- and tri-methylated lysines and arginines, are best characterized. Tri-methylation of lysine 27 of histone 3 (H3K27me3) was shown to be generally associated with a repressive chromatin state and has been shown to be crucial for vernalization in cereals and other plants (Dennis and Peacock 2009; Diallo et al. 2012; Oliver et al. 2009). Another histone tail modification, tri-methylation of lysine 4 of histone 3 (H3K4me3), correlates with gene activation in cereals (Dong and Weng 2013; Liu et al. 2015), similarly as observed in other animal and plant species. Chromatin modulators such as small RNA-related pathways (siRNAs and miRNAs), which act directly on chromatin and induce RNA-dependent DNA methylation (RdDM), have also been described (Lanciano and Mirouze 2017). In plants, sRNAs are classified into several major classes, including microRNAs (miRNAs), Piwi-interacting RNAs (piRNAs), small interfering RNAs (siRNAs), which enclose phased and secondary interfering RNAs (phasiRNAs), small nucleolar RNAs (snoRNAs), tRNA-derived small RNAs (tsRNAs), small rDNA-derived RNA (srRNAs), and small nuclear RNA (U-RNAs) (Asensi-Fabado et al. 2017; Fei et al. 2013). Among these, miRNAs generated from dsRNA regions of hairpin-shaped precursors, and siRNAs, including phasiRNAs, originating from long dsRNAs (Asensi-Fabado et al. 2017; Fei et al. 2013; Kouzarides 2007), are the best studied in cereals and other plant species. How these pathways control reproductive development in cereals and how they ultimately respond to reduce stress effects and still generate seeds, is a major topic of this review.

During reproductive development, most epigenetic studies have been conducted at well-defined and discrete stages. Here, we are focusing on three developmental stages during reproductive development and how they are affected by environmental stresses. First, we describe (1) epigenetic alterations during pre-fertilization processes (male and female gametogenesis). Then, we discuss (2) fertilization mechanisms, gamete activation, and early seed development involving pro-embryo and syncytium formation as well as endosperm cellularization. Finally, we cover a stage known as (3) seed maturation, in which starch accumulation, desiccation, and seed dormancy are major processes taking place. Even though reproductive development can be subdivided into several other stages, recent reports have shown the importance of the aforementioned stages or phases as determinant for productivity in cereals (Begcy and Walia 2015a, b; Chen et al. 2016; Folsom et al. 2014; He et al. 2015; Lamaoui et al. 2018; Muller and Rieu 2016; Parihar et al. 2015; Sabelli 2012; Sade et al. 2018), especially when exposed to abiotic stresses. We highlight recent advances and provide an integrated model of epigenetic regulation incorporating the main key players controlling reproductive development described so far in cereals under abiotic stresses.

## Abiotic stresses affect pollen development

Pollen formation is a major aspect of plant reproduction. Several genes and epigenetic factors controlling cell fate during gametophyte formation have been described in cereals. Male germline development in maize is regulated by the glutaredoxin gene *male sterile converted anther 1* (*MSCA1*), which promotes male germline cell fate and meristem growth (Chaubal et al. 2003). Moreover, it was shown that during stress-associated hypoxia, a common condition in rice fields due to flooding, low levels of oxygen stimulate the increase in germ cell numbers, thus promoting ectopic formation of germ cells in the epidermal layer. Conversely, oxidizing environments inhibit germ cell specification and cause ectopic differentiation in deeper tissues as well as a reduction in archesporial cell number, which are progenitors of the germline (Kelliher and Walbot 2012). In the *msca1* mutant 24-nt phased small interfering RNAs (phasiRNAs) were lacking, suggesting that stress-induced mis-regulation of *MSCA1* causes the absence of phasiRNAs in anthers and thus affects a number of unknown target genes (Zhai et al. 2015). Another key regulator during male germline development, that is oxygen dependent, is multiple archesporial cells 1 (*MAC1*). When *MAC1* is expressed, a small protein is generated that induces adjacent cells to differentiate into supportive tissues (Wang et al. 2012). Anthers submitted to heat stress in barley (*Hordeum vulgare*) showed gradual degeneration of epidermal and archesporial cells resulting in anther abortion as a consequence of transcriptional inhibition (Abiko et al. 2005). Reduced *MAC1* expression might be a cause for this observation. However, it seems possible that *MAC1* is regulated by chromatin modifications (Table 1).

**Table 1:** Genes regulated in response to abiotic stress during reproductive development in cereals

Epigenetic mechanisms	Genes affected	References
*Male gametogenesis*		
phasiRNAs	Male sterile converted anther 1 (*MSCA1*)	Chaubal et al. (2003), Kelliher and Walbot (2012)
H3K9me2/H3k9ac/H3S10phos/phasiRNAs	Meiosis Arrested at Leptotene 1 (*MEL1*)	Ding et al. (2012), Komiya et al. (2014)
H2A phosphorylation	Bub1-related kinase 1 (*BRK1*)	Wang et al. (2012)
miRNAs	Suppressor of G_2_ allele of skp1 (*SGT1*)	Liu et al. (2017)
*Female gametogenesis*		
DNA methylation; histone acetylation	ABA-related genes	Tang et al. (2008), (2014)
DNA methylation	DNA methyltransferases (*DMTs*)	Zhuang et al. (2008)
*Early seed development*		
H3K4me3/H3K27me3	Polycomb repressive complex 2 (*PRC2*)	Folsom et al. (2014)
DNA methylation	MADS-box protein (*MADS87*)	Folsom et al. (2014), Chen et al. (2016)
DNA methylation	MADS-box protein (*MADS69*)	Folsom et al. (2014), Chen et al. (2016)
DNA methylation	MADS-box protein (*AGL36*)	Folsom et al. (2014), Chen et al. (2016)
H3K4me3	Alcohol dehydrogenase (*ADH*)	Tsuji et al. (2006)
DNA methylation	PINFORMED1-mediated transport of auxin (*PIN1*)	Zhao et al. (2017)
DNA methylation	Miniature inverted-repeat transposable elements (*MITEs*)	Li et al. (2014)
*Seed maturation*		
DNA methylation	ABA- and GA-related genes	Begcy and Walia (2015a, b), Xing et al. (2015)
DNA methylation	Demeter (*DME*)	Kapazoglou et al. (2013)
Histone acetylation	ADP-glucose pyrophosphorylase (*AGPS2*)	Zhao et al. (2014), Wang et al. (2017)
DNA methylation	Protein phosphatase 2C (*PP2C*)	Liu et al. (2015), Yu et al. (2016)
DNA methylation	SNF1-related protein kinase 2 (*SNF1*)	Liu et al. (2015), Yu et al. (2016)
DNA methylation	ABA insensitive 5 (*ABI5*)	Liu et al. (2015), Yu et al. (2016)
DNA methylation	Lipid phosphate phosphatase 2 (*LPP2*)	Liu et al. (2015), Yu et al. (2016)
DNA methylation	Auxin response factor (*ARF*)	Liu et al. (2015), Yu et al. (2016)

Four major reproductive processes are distinguished. The underlying epigenetic mechanisms, affected genes, and corresponding references are indicated

During male gametophyte formation in rice, it was reported that transition of pollen mother cells (PMCs) from pre-meiosis to meiosis stage is controlled by an increase in di-methylation of lysine 9 of histone 3 (H3K9me2) and a decrease in acetylation of lysine 9 of histone 3 (H3K9) as well as phosphorylation of histone 3 (H3S10) (Liu and Nonomura 2016). These well-coordinated epigenetic events regulate *Meiosis Arrested At Leptotene 1* (*MEL1*), which encodes a germline-specific AGO protein that preferentially binds 21-nt phasiRNAs derived generally from intergenic regions. Additionally, *MEL1* plays a central role in the progression of meiosis beyond the leptotene stage (Komiya et al. 2014). Rice *mel1* mutants show abnormal tapetum and aberrant vacuolation of microspore mother cells impeding chromosome condensation during early meiosis, thus causing male sterility (Nonomura et al. 2007). This indicates that accumulation of 21-nt phasiRNAs induces male sterility. Moreover, new reports identified lncRNAs, which are successively processed into sRNAs as main regulators of male sterility in rice (Ding et al. 2012). It was further supported that alterations in photoperiod and temperatures cause single spontaneous mutations in sRNAs leading to male sterility in rice (Fan and Zhang 2018; Zhou et al. 2012).

In maize, phasiRNA biogenesis shows a high level of spatiotemporal dynamics throughout anther development (Zhai et al. 2015) with preferential accumulation of 21-nt phasiRNAs during pre-meiosis and 24-nt phasiRNAs during meiosis. The latter persist during pollen formation. Additionally, a stage-specific study in maize anthers at meiosis stage, particularly during zygotene, also reported abundant 21- and 24-nt phasiRNAs as well as two novel miRNAs, zma-MIR11969 and zma-MIR11970 (Dukowic-Schulze et al. 2016). Remarkably, higher levels of DNA methylation at phasiRNAs loci were found especially in the CHH context indicating a putative role of phasiRNAs in cis-DNA methylation. Targets for 24-nt phasiRNAs have not yet been identified during pollen development. However, in maize Argonaute (*ZmAGO18b*) was pointed out as a possible candidate based on its transcriptional profile (Fei et al. 2016). Further experimental evidence is required to confirm this finding. During heat and oxidative stress, wheat phasiRNAs and sRNAs were reported to be highly up-regulated (Wang et al. 2016). Since phasiRNAs and other sRNAs regulate male gametophyte development, it was suggested that their mis-regulation due to increased temperatures and the presence of other stresses such as drought causes pollen malformation. This is commonly observed in mutants of genes controlled by sRNAs as well as under abiotic stress conditions.

In drought-stressed wheat plants, it has been shown that increased abscisic acid (ABA) concentration in pollen grains correlates well with pollen sterility and decreased yield (Ji et al. 2011; Parish et al. 2012). Notably, ABA was also significantly higher in rice florets exposed to heat stress (Tang et al. 2008), indicating that an overall increase in ABA in reproductive tissues has negative effects in plant reproduction. In rice plants drought-stressed during the pollen meiosis stage, hormone pathways related to gibberellic acid signaling and abscisic acid catabolism were shown to be reprogrammed (Jin et al. 2013). Nevertheless, detailed hormonal analyses linked with transcriptomic and epigenetic studies are needed to shed light on the role of ABA and other hormones in reproductive tissues exposed to abiotic stresses. Recently, an interesting approach integrating genome-wide sRNA analysis with quantitative trait locus (QTL) mapping in rice identified four major QTLs for heat tolerance during flowering (*qHT*-*3, qHT*-*6, qHT*-*8,* and *qHT*-*12*). A detailed analysis of QTLs revealed the presence of miRNA targets associated with ABA-responsive genes. Additionally, suppressor of the G_2_ allele of *skp1* (*SGT1*), a direct target of miRNA166e, was also mapped within the *qHT*-*8* locus (Liu et al. 2017). SGT1 has been pointed out as a positive regulator of thermal responses by interacting with heat shock protein (HSP) 90 and HSP70 (Gorovits and Czosnek 2017; Park and Seo 2015). These findings suggest the existence of an integrative mechanism of miRNAs and their targets on modulating abiotic stress responses during anther and pollen development. Finally, even though recent reports have highlighted the role of DNA methylation and sRNAs including phasiRNAs and miRNAs during abiotic stress responses on male germline development, further research addressing the role of other sRNAs and chromatin remodeler genes is needed.

## Abiotic stresses during embryo sac development

Although the impact of abiotic stresses on female gametophyte development has been noticed since decades, due to their inaccessibility, little is known about how stresses impact this important developmental tissue. Water deficit induced an increase in the number of antipodal cells in the maize embryo sac, while the growth and receptivity of the style were inhibited (Ribaut et al. 2009). Additional observations pointed toward a significant delay in female organ development of drought-stressed maize plants (Damptey and Aspinall 1976; Damptey et al. 1978). Elevated ABA concentration in ovaries was suggested as the main cause of induction of female flower abortion in stressed plants (Asch et al. 2001). The role of ABA in mediating gene expression changes in response to abiotic stresses is well known (Chinnusamy et al. 2008; Hyun et al. 2017; Wong et al. 2017). In rice, histone deacetylation was observed in plants submitted to cold, mannitol, and salt stress, resulting in repression of ABA regulatory genes. Histone deacetylation imposes a non-permissive chromatin conformation leading to transcriptional repression (Fu et al. 2007). Interestingly, drought stress induced a raise in the ABA concentration in developing ovules of maize and wheat (Andersen et al. 2002; Ji et al. 2011; Kakumanu et al. 2012). This has been suggested to impair cell division, potentially affecting fertilization and further seed formation (Yang et al. 2001). A recent study analyzing whole-genome DNA methylation in rice female gametes (egg and central cell) indicates that under non-stress conditions both cell types display similar methylation patterns compared to other tissues (Park et al. 2016). Whether this pattern changes during stress application has not been investigated. A decrease in global methylation has been reported under stress conditions in rice and ryegrass (Karan et al. 2012; Tang et al. 2014). It was suggested that elevated ABA concentrations reported in earlier studies could possibly be the result of reduced DNA methylation of hormone-related genes. However, these could also be an indirect effect, since new findings in Arabidopsis suggest a low correlation between changes in DNA methylation and transcriptional regulation (Kawakatsu et al. 2016; Meng et al. 2016).

## Do abiotic stresses influence fertilization mechanisms?

Before fertilization is executed in cereals, pollen grains are deposited by wind or animals at papilla hairs of feathery stigmata (silks in maize) where they germinate and grow toward the ovule to deliver its sperm cell cargo. To which extent this so-called progamic phase of reproduction is affected by abiotic stresses is almost completely unclear. A comparison between pre- and post-pollinated silks in maize identified 30 conserved miRNA families (Li et al. 2013). Notably, all families have been associated previously with stress responses. For instance, miR169, miR171, miR393, miR395, and miR528 have been correlated with drought and salt stress responses (Ferreira et al. 2017; Zhao et al. 2009). These findings indicate that pollination and stress-related processes are closely associated and might be genetically regulated by similar epigenetic mechanisms. After sperm cell release, gametes are activated, fusion between the two sperm cells and female gametes takes place, and both embryo and endosperm development stages are initiated (Dresselhaus et al. 2016). Whether these processes, which occur deeply embedded and protected within the maternal tissues of both ovule and ovary, are affected by stress factors, is not known. At present, we understand little about these events under non-stress conditions. It has been controversially discussed whether gene expression in both embryo and endosperm is largely maternal in origin until three to four days after fertilization. It was further suggested that this is epigenetically regulated (e.g., Baroux and Grossniklaus 2015; Grimanelli et al. 2005; Nodine and Bartel 2012; Zhao et al. 2017; Zhao and Sun 2015). However, recent evidence using genome-wide transcriptional analysis in maize and rice revealed that zygotic genome activation occurs soon after fertilization, exhibiting a highly dynamic and partially transient pattern of gene expression (Chen et al. 2017; Anderson et al. 2017). These include genes involved in hormone biosynthesis and responses, RdDM, and other pathways associated with massive chromatin changes after fertilization. Upon fertilization, for example, several genes encoding histone H3 variants, that are expressed at low levels in male and female gametes, are activated 12 h after fertilization together with H1, whose transcripts are absent in sperm cells (Chen et al. 2017). Overall, these patterns indicate that fertilization and early seed development might be very vulnerable to abiotic stresses, but this remains to be demonstrated.

Under heat stress, a strong reduction in fertility was observed when male and female gametes were stressed prior to fertilization. In contrast, cold-stressed gametes did not show significant differences in fertilization rates (Dupuis and Dumas 1990). These observations suggested that high temperatures impose a strong pressure on fertilization. For instance, increased temperatures can decrease the time in which female reproductive structures such as feathery stigmata of cereals are more receptive to pollen, thereby reducing the chances for a successful fertilization. In maize, pollen–stigma interaction and early kernel development were dramatically affected by heat stress, resulting in a significant reduction in kernel formation (Mitchell and Petolino 1988). Further systematic research approaches are required to investigate the molecular mechanisms affected and to distinguish between fertility effects caused by defective gametophytes/gametes or processes directly affecting fertilization-related processes.

## Abiotic stresses strongly affect early seed development

Early seed development is a highly organized and synchronized genetic process that leads to the formation of both the embryo and the endosperm. In contrast to dicotyledonous plants, where little remnants of endosperm are present at seed maturity, a persistent endosperm in cereals stores starch and proteins required for seedling growth during germination (Hands et al. 2012). Although mature seeds of dicotyledonous and monocotyledonous plants are different regarding complexity of embryo development and endosperm content, initial events during early seed development appear partly similar (Sabelli 2012). Abiotic stresses during early seed development are major limiting factors for cereals production (Begcy and Walia 2015a, b; Chen et al. 2016; Folsom et al. 2014; Hyun et al. 2017). During stress conditions at this specific reproductive stage, cereals exhibit limited endosperm proliferation affecting final seed size (Fujita et al. 2013; Kapazoglou et al. 2010; Li et al. 2015; Luhua et al. 2013). For instance, heat- and drought-induced losses in barley and wheat have contributed to approximately a 60% decrease in grain weight as well as in seed size (Begcy and Walia 2015a, b; Ejaz and von Korff 2017). Particularly, environmental stresses delay developmental transition from syncytial to cellularization stage of endosperm development. Coincidently with reduced seed size and delayed endosperm development, a subset of genes associated with cell cycle, chromatin assembly, cytoskeleton- and microtubule-related processes as well as hormone balance have been recurrently found as being differentially regulated by environmental stresses during seed development (Begcy and Walia 2015a, b). Concomitantly, reduced protein levels of these genes have been reported under stress conditions (Kesten et al. 2017; Zhang et al. 2017). Mutations and overexpression of some of the genes resulted in a delay or failure of transition toward cellularized endosperm (Dante et al. 2014; Yi et al. 2011).

**Fig. 1:**
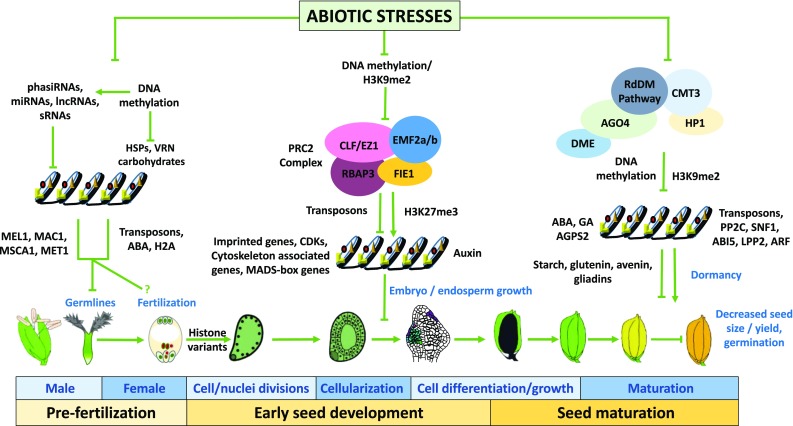
Integrative model of the effects of abiotic stresses on the epigenetic status of cereals during reproductive development. Pre-fertilization, early seed development and maturation events in response to stresses are described. The main epigenetic alterations and corresponding molecular players are indicated. See text for detailed explanations

At the epigenetic level, it was shown that polycomb repressive complex 2 (PRC2) represents a major regulator of early seed development. During endosperm development in cereals, several active members of PRC2 have been identified (Kapazoglou et al. 2010; Tonosaki and Kinoshita 2015). Depending on protein sub-unit combinations of PRC2, several distinct roles have been attributed during seed formation. In barley, two members of the PRC2 complex, fertilization-independent endosperm (FIE) and enhancer of zeste (E(z)), were shown to affect seed size by regulating endosperm development (Kapazoglou et al. 2013). In rice, repression of FIE1 resulted in abnormal endosperm formation, strongly supporting the idea that PRC2 regulates endosperm development in cereals through DNA methylation and histone modifications, similar to findings in the model plant Arabidopsis. Rice plants submitted to heat stress during early seed development had a significant reduction in seed size (Folsom et al. 2014). This decrease was correlated with mis-regulation of *FIE1* expression levels. In cereals, *FIE1* is the only member of the PRC2 complex described so far with an endosperm-specific expression pattern (Folsom et al. 2014; Kapazoglou et al. 2010; Tonosaki and Kinoshita 2015). The *FIE1* promoter is methylated in the sperm cell, but not in the central cell. After fertilization, an asymmetric pattern of parental DNA methylation is inherited by the endosperm in cereals and other plants. Paternally derived *FIE1* is silenced, while maternally derived alleles are expressed. However, after heat stress both paternal and maternal *FIE1* alleles are strongly repressed during early seed development (Chen et al. 2016; Folsom et al. 2014). Together with *FIE1* several MADS-box genes including *OsMADS87, OsMADS69* and *OsAGL36* were mis-regulated in heat-stressed rice seeds, which led to a decrease in yield (Chen et al. 2016; Folsom et al. 2014). In barley, heat stress also triggered an altered expression of MADS-box genes (Ejaz and von Korff 2017), suggesting that PRC2-mediated regulation may be sensitive to heat stress and could affect genes typically active during early stages of endosperm development (Fig. 1). Another type of epigenetic control was observed in rice plants submitted to submergence, in which H3K4me3-mediated regulation of alcohol dehydrogenase (*ADH*) expression was regulated in a biphasic manner. First, levels of histone H3K4 changed from a di- to a tri-methylation state followed by an increase in acetylation of H3 (Tsuji et al. 2006). In contrast to H3K27me3, which is associated with gene silencing, H3K4me3 typically correlates with transcriptional activation (Zhang et al. 2007). A global DNA methylation study during seed development in rice showed that the expression of genes responding to the growth hormone auxin and the stress hormone ABA was controlled by DNA methylation (Xing et al. 2015). Recently, this has also been described in barley (Surdonja et al. 2017), indicating that the cellularization process of cereal endosperm could be especially stress-sensitive. Therefore, it was suggested that DNA methylation controls ABA levels and thus promotes endosperm cellularization in cereals. Alteration in embryo and endosperm formation during stress could thus be due to a combined alteration of PRC2 regulation and hormonal imbalance, resulting, as a consequence, in alteration in gene expression. In summary, the combination of hormonal and epigenetic regulation through DNA methylation as well as H3K27me3 and H3K4me3 methylation appears critical during early seed development and thus might be especially sensitive to environmental stresses.

Reactivation of TEs can occur under certain situations including environmental conditions or after genome rearrangements (Vicient and Casacuberta 2017). In rice, heat stress during early seed development triggered genome-wide alterations including reactivation of transposable elements by a decrease in DNA methylation at noncoding regions (Chen et al. 2016). This particular activation was more accentuated with the severity of the stress indicating a temperature-sensitive quantitative response (Chen et al. 2016). Elevated rates of retrotransposon movement creating new insertional polymorphisms, which are associated with deleterious effects, were indeed induced by microclimate changes in barley and rice (Kalendar et al. 2000; Naito et al. 2006). Therefore, mis-regulation of DNA methylation by increased temperatures results in changes in the epigenetic state of the genomes, which likely cause a significant rearrangement of TE activity.

## Abiotic stresses during seed maturation

Seed maturation is a key stage during seed development in which fundamental processes including embryo growth and enlargement terminate, while mechanisms leading to accumulation of storage products, desiccation tolerance and seed dormancy are activated. In heat-sensitive wheat varieties, it was shown that heat stress applied during the seed maturation stage strongly affected seed vigor and germination rate by changing, for example, the amount of storage products and hormones (Hasan et al. 2013). In contrast, a 24-h period of moderate heat stress in rice during the same stage generated seeds that exhibited better and faster germination rates compared to non-stressed seeds. More detailed characterization of heat-stressed seeds showed that a short-term period of stress resulted in higher hormonal status of gibberellic acid (GA) and ABA, as well as of total starch content. These findings indicate that a 24-h post-zygotic heat stress potentially primes embryos of some lines to germinate faster, possibly through an altered sensitivity to ABA and increased resource allocation. Nonetheless, any longer period of heat stress was detrimental for seed establishment and germination (Begcy and Walia 2015b). Additional evidence also indicates that active DNA demethylation triggered by stresses altered regulation of ABA synthesis genes resulting in higher ABA levels (Kakumanu et al. 2012; Xing et al. 2015). A similar response was observed in heat-stressed rice and drought-stressed wheat seeds, in which a subset of genes associated with cytoskeleton organization and hormone pathways were mis-regulated. Notably, strong transcriptional repression was also observed in genes associated with wheat storage proteins such as gliadins, glutenins, and avenins (Begcy and Walia 2015a, b; Chen et al. 2016). Notably, both the aforementioned pathways have been shown to be controlled by PRC2 (Kapazoglou et al. 2010; Tonosaki and Kinoshita 2015), indicating that likely PRC2-mediated regulation also plays an important role during seed maturation under drought stress (Fig. 1).

In barley, a DNA glycosylase closely related to Demeter (DME) required for maternal allele demethylation and imprinting in the endosperm was reported to be involved in responses to stress. In a drought-tolerant barley variety, increased *HvDME* expression was correlated with enhanced tolerance compared with a drought-sensitive variety. Variation in tolerance between varieties was partially explained by different levels of DNA methylation found in specific regions of the promoter and gene body of *HvDME* (Kapazoglou et al. 2013). Additionally, the presence of a *Copia* retrotransposon element within the 3′ downstream region of *HvDME* might have contributed to increased tolerance, although the detailed molecular mechanisms remained unclear. Similarly, enhanced tolerance to abiotic stresses has also been described recently in common wheat by a miniature inverted-repeat transposable elements (MITEs) insertion into the 3′-UTR of HSP16.9 affecting its transcription rate (Li et al. 2014; Makarevitch et al. 2015). This indicates that transposon activity might have significantly contributed to the establishment of genetic responses to abiotic stress.

A recent study performed during rice seed development detected a strong increase in acetylation levels of proteins during seed differentiation and maturation (Wang et al. 2017). Most acetylated proteins were associated with processes involved in starch and sucrose metabolism, glycolysis/gluconeogenesis, and the tricarboxylic acid (TCA) cycle. This pattern of acetylation coincides with the starch biosynthesis phase in rice, which begins at 5 DAF (days after fertilization) and continues through seed maturation. During/after abiotic stress, a high induction of acetylation was also found at a genome-wide context at histone proteins. During stress conditions, the promoter region of cell cycle genes showed hyper-acetylation at specific lysine sites at H3 and H4 tails in maize, which correlated with prolonged cell cycle duration and an inhibitory effect of growth and development (Zhao et al. 2014). However, one cannot exclude the possibility that increased acetylation of cell cycle genes might be an indirect effect of abiotic stresses. Thus, it is important to highlight that besides the known role of DNA methylation, histone acetylation also controls important transition phases during seed development in cereals and thus opens the door for further studies on how acetylation and other posttranscriptional modifications are altered and affected by abiotic stresses during seed development.

Additionally, RdDM pathways have been shown to regulate many developmental processes including seed maturation in cereals (Lanciano and Mirouze 2017; Liu et al. 2016). Methylated histones recruit a specific DNA methyltransferase, chromomethylase 3 (CMT3), through physical interaction with heterochromatin protein 1 (HP1) to the DNA and trigger cytosine methylation (Dangwal et al. 2014; Smallwood et al. 2007). In barley and wheat, AGO proteins, which participate in gene silencing through RdDM pathways and which are components of the RNA-induced silencing complex (RISC), were shown to regulate seed maturation and dormancy (Singh and Singh 2012; Singh et al. 2014). When barley plants were submitted to terminal stress during grain filling, increased DNA methylation was observed, for example, in the promoter region of the *Cytokinin*-*Oxidase 2.1* (*CKX2.1*) gene, which is a target of the RdDM pathway (Surdonja et al. 2017). Moreover, it was reported that dormancy was reduced at both increased temperatures and water deficit during seed maturation (Begcy and Walia 2015b; Singh et al. 2014). Regulation of dormancy is associated with a decrease in seed sensitivity to signaling molecules including hormones such as ABA and auxin (IAA). Their biosynthesis and readout were shown to be affected as several hormone-related genes encoding, for instance, protein phosphatase 2C (PP2C), SNF1-related protein kinase 2 (SNF1), ABA insensitive 5 (ABI5), lipid phosphate phosphatase 2 (LPP2), and auxin response factors (ARFs), which are transcriptionally repressed during stress exposure, were mis-regulated (Liu et al. 2013; Yu et al. 2016). It is thus likely that initially RdDM pathways are affected, which then cause mis-regulation of the above-mentioned genes. However, this correlation leading to shortening of dormancy under stress conditions requires further experimental evidence.

## Conclusions and perspectives

Epigenetic mechanisms controlling reproductive development have been shown in the past decade as pivotal regulators of stress responses in cereals. Development and function of female and especially male gametophytes appear to be especially susceptible to abiotic stresses. DNA methylation and small regulatory RNAs including phasiRNAs, lncRNAs, and miRNAs have been shown as repressors of germline initiator genes. Mis-regulation of germline-specific genes during abiotic stresses seems to be a consequence of the lack of the aforementioned epigenetic regulators (Fig. 1).

It is still unclear whether the fertilization process itself is affected by stresses and if so, which epigenetic components are the main targets. However, since histone variants are exchanged during zygotic gene activation at non-stress conditions and DNA methylation of imprinted genes is active immediately after fertilization, we speculate that this stage is also affected by abiotic stresses. Proper activity of the PRC2 complex is very critical for seed formation during early stages involved in cell/nuclei divisions, endosperm cellularization, and embryo patterning (Fig. 1). Abiotic stresses such as drought and heat stress affect the expression of PRC2 complex members by removing their H3K9me3 epigenetic marks. DNA methylation of developmental regulator genes including MADS-box genes repressed by PRC2 under non-stress conditions is reduced by abiotic stresses leading to a shift in their transcriptional expression and thus to seed malformation and/or a delay in development (Fig. 1). During seed maturation, hormone levels of ABA, GA, and IAA are controlled by DNA methylation. Changes in their levels under stress conditions strongly impact proper seed development. Together with epigenetic factors including the RdDM pathway, which recruits AGO, CMT3, DME, and other proteins, hormone imbalance represents a key regulator of stress responses.

These above-described findings now provide exciting prospects since a large number of these processes are crucial for maintenance of yield and reproductive success under abiotic stress conditions. In conclusion, even though plants are exposed to abiotic stresses throughout their life cycle, reproductive development is more vulnerable to abiotic stresses compared to vegetative developmental stages as stress may terminate development and lead to complete sterility. The systematic study of epigenetic regulatory mechanisms under abiotic stress conditions during reproductive development needs to be extended and approached under field conditions, since multiple stresses are frequently coexistent. Inheritable epigenetic changes such as small regulatory mechanisms, histone variants, and DNA methylation are also unexplored in cereals in a trans-generational memory context, since these epigenetic variations might improve stress resilience in the offspring.

Finally, although new tissue- and cell-specific methodologies including fluorescence-activated cell sorting, ATAC-seq, and methylome sequencing (e.g., Begcy and Dresselhaus 2017; Borges et al. 2012; Buenrostro et al. 2015; Farlik et al. 2015) permit to study reproductive development in spatiotemporal detail in response to stresses, some key developmental processes such as embryo sac development and fertilization still remain largely unexplored due to technical difficulties associated with their isolation and observation. Further advances in the sensitivity of methods allowing single cell genome sequencing, interactome, proteome, and metabolome studies as well as gene-editing technologies, which work well for cereals, will facilitate systematic and comprehensive genome-wide studies and the identification of new candidate genes controlling epigenetic responses in cereal to environmental stresses.
